# Antibody levels following booster SARS-CoV-2 vaccination among dialysis patients initially vaccinated with adenovirus vector-based vaccine

**DOI:** 10.1007/s40620-022-01559-8

**Published:** 2023-01-23

**Authors:** Joanna Willetts, Linda H. Ficociello, Curtis D. Johnson, Sandra E. Alexander, Claudy Mullon, Jeffrey L. Hymes

**Affiliations:** 1 Global Medical Office, Fresenius Medical Care, Waltham, MA USA; 2 Spectra Laboratories, Southaven, MS USA; 3 Fresenius Medical Care, 1000 Corporate Centre Drive, Suite 400, Franklin, TN 37067 USA

**Keywords:** Dialysis, COVID-19, Vaccination, Antibody, mRNA vaccine, Ad26.COV2.S

In the United States, three vaccines directed against severe acute respiratory syndrome coronavirus 2 (SARS-CoV-2) have been used in clinical practice: two messenger RNA-based vaccines (BNT162b2 and mRNA-1273) and one adenovirus vector-based vaccine (Ad26.COV2.S). Data suggest that dialysis patients demonstrate diminished reactivity to vaccination with Ad26.COV2.S relative to mRNA-based vaccination, as assessed by magnitude and duration of antibody response [1, 2]. Although booster vaccination, preferably with an mRNA-based vaccine, is recommended, the effect of booster doses among dialysis patients who initially received an adenovirus-based vaccine has not been characterized.

As part of a continuing quality-improvement project across Fresenius Kidney Care (FKC) clinics, we examined antibody levels among dialysis patients who received primary vaccination against COVID with Ad26.COV2.S. Patients who received no additional vaccine doses, those who received an additional dose of Ad26.COV2.S, and those who received one to two doses of an mRNA vaccine were included in the present analysis. Patients with laboratory evidence of COVID-19 infection (positive polymerase chain reaction test) and those with suspected or documented COVID-19 infection were excluded.

Antibody levels were assessed in remnant blood samples at a centralized laboratory using a semiquantitative chemiluminescent assay for immunoglobulin G directed against the receptor-binding domain of the S1 subunit of the SARS-CoV-2 spike antigen (ADVIA Centaur^®^ XP/XPT sCOVG; Siemens Healthcare Diagnostics Inc.; Tarrytown, NY). Antibody indices were broadly categorized as nonreactive (< 1) or reactive (≥ 1). Reactive indices were further categorized as adequate (> 7) and beyond the maximum detectable range (≥ 750) [1]. Patients were included in the present analysis if they had at least two antibody tests available: one collected after administration of the initial Ad26.COV2.S vaccination and before eligibility for an additional vaccine dose at approximately 6 months (i.e., early antibody level), and one collected after availability/eligibility of additional vaccine doses (i.e., late antibody level). For patients with multiple antibody levels during a given time period, the last value was included in the analysis. All patients allowed the use of their remnant blood samples collected for routine care for research purposes as part of the consent form signed upon receiving treatment.

Overall, 839 patients receiving dialysis at FKC clinics received primary vaccination with Ad26.COV2.S. After excluding those with vaccination regimens that could not be determined (*n* = 2), those with prior COVID infection (*n* = 281), and those without early and late antibody levels (*n* = 102), the analysis cohort included 454 patients. Approximately 23% of patients (*n* = 103) did not receive additional vaccination after the initial adenovirus-based vaccination (Ad26.COV2.S). Of the 351 patients receiving additional vaccine doses, 8 (2%) received a second dose of the adenovirus-based vaccine (Ad26.COV2.S + Ad26.COV2.S), 329 (72%) received a single dose of an mRNA vaccine (Ad26.COV2.S + mRNA), and 14 (3%) received 2 doses of an mRNA vaccine (Ad26.COV2.S + mRNA + mRNA). Patients who received a second vaccine dose did so a median of 231 (Ad26.COV2.S + mRNA + mRNA) to 243 (Ad26.COV2.S + Ad26.COV2.S) days after their primary vaccination.

**Fig. 1:**
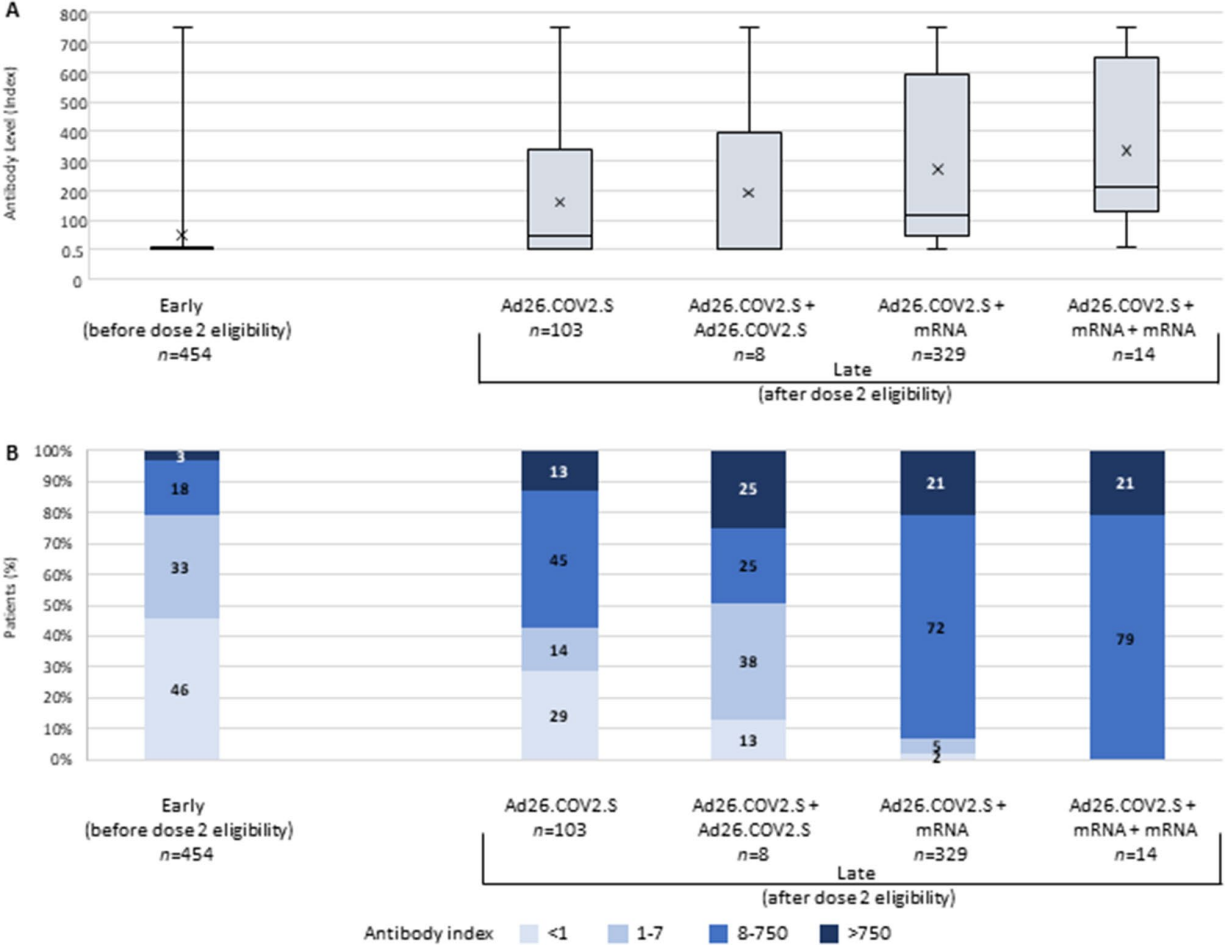
Antibody response after the first dose of Ad26.COV2.S and after eligibility for additional doses. In the box plot (**A**), the range (error bars), interquartile range (rectangle), median (horizontal line), and mean (*x*) antibody indices are depicted. Categorical results are presented in graph **B**. When more than one measurement was available during a given time period, the latest value was used. Measurements < 0.5 and > 750 index were included in the calculation as 0.5 and 750, respectively. For the “early” group, the assessment was completed a median of 96 days after primary vaccination. For the “late” antibody assessments, the last follow-up assessment was completed a median of 327 days after the primary vaccination for the Ad26.COV2.S group, a median of 89 days after the booster in the Ad26.COV2.S + Ad26.COV2.S and Ad26.COV2.S + mRNA groups, and a median of 21 days after the second booster in the Ad26.COV2.S + mRNA + mRNA group

Based on early antibody levels (mean [SD] of 97 [12] days after initial vaccination), 46% of patients were considered nonreactive and fewer than 25% had adequate antibody levels. Late antibody levels were assessed, on average (SD), 325 (14) days after initial vaccination. Median antibody indices were markedly higher among patients who received additional vaccination with an mRNA vaccine (Fig. 1A). Antibody indices ≤ 7 were observed for 43% and 51% of the Ad26.COV2.S and Ad26.COV2.S + Ad26.COV2.S subgroups, respectively. In contrast, only 7% of patients receiving one booster of an mRNA vaccine exhibited an inadequate late antibody response. All patients receiving two mRNA booster doses exhibited adequate antibody responses.

Our findings should be reviewed in the context of several methodological limitations. Although we aimed to exclude patients infected with SARS-CoV-2, it is likely that not all cases were excluded, as suggested by the increase in mean (SD) antibody titers from the early measurement to the late measurement among patients who did not receive any additional vaccine dose (48 [147]–177 [253]). This may be attributable to an increase in asymptomatic infection. We do not have consistent documentation of reasons for not receiving boosters. Our sample size for some vaccination combinations were small. We also cannot generalize differences in antibody levels to differences in clinical effectiveness. Brunelli and colleagues recently published data suggesting primary vaccination with either Ad26.COV2.S or BNT162b2 exhibited similar effectiveness at 6 months among dialysis patients [3].

Our findings that booster vaccine doses enhance serologic immune responses among dialysis patients are consistent with findings from cohorts initially treated with mRNA vaccines [4]. Our findings are unique in that we demonstrated a greater immune response among those patients receiving a heterologous vaccine regimen (i.e., adenovirus- and mRNA-based vaccines) than those receiving a homologous regimen (adenovirus-based only). In contrast, Reindl-Schwaighofer et al. found similar benefit in administering Ad26.COV2.S vs. mRNA vaccines to kidney transplant recipients previously receiving two doses of an mRNA vaccine [5]. Our results support the practice of administering booster doses of mRNA vaccines for all eligible dialysis patients who received primary vaccination with adenovirus-based vaccines.
